# Mechanostimulation of breast myoepithelial cells induces functional changes associated with DCIS progression to invasion

**DOI:** 10.1038/s41523-022-00464-4

**Published:** 2022-09-20

**Authors:** Mary-Kate Hayward, Michael D. Allen, Jennifer J. Gomm, Iain Goulding, Clare L. Thompson, Martin M. Knight, John F. Marshall, J. Louise Jones

**Affiliations:** 1 Centre for Tumour Biology, Barts Cancer Institute, Queen Mary University of London, John Vane Science Centre, Charterhouse Square, London, EC1M 6BQ UK; 2 School of Engineering and Materials Science, Queen Mary University of London, Mile End Road, London, E1 4NS UK

**Keywords:** Breast cancer, Predictive markers, Growth factor signalling

## Abstract

Women with ductal carcinoma in situ (DCIS) have an increased risk of progression to invasive breast cancer. Although not all women with DCIS will progress to invasion, all are treated as such, emphasising the need to identify prognostic biomarkers. We have previously shown that altered myoepithelial cells in DCIS predict disease progression and recurrence. By analysing DCIS duct size in sections of human breast tumour samples, we identified an associated upregulation of integrin β6 and an increase in periductal fibronectin deposition with increased DCIS duct size that associated with the progression of DCIS to invasion. Our modelling of the mechanical stretching myoepithelial cells undergo during DCIS progression confirmed the upregulation of integrin β6 and fibronectin expression in isolated primary and cell line models of normal myoepithelial cells. Our studies reveal that this mechanostimulated DCIS myoepithelial cell phenotype enhances invasion in a TGFβ-mediated upregulation of MMP13. Immunohistochemical analysis identified that MMP13 was specifically upregulated in DCIS, and it was associated with progression to invasion. These findings implicate tissue mechanics in altering the myoepithelial cell phenotype in DCIS, and that these alterations may be used to stratify DCIS patients into low and high risk for invasive progression.

## Introduction

For the majority of invasive breast cancers, progression follows transition through the pre-invasive stage of ductal carcinoma in situ (DCIS)^1^. In DCIS, tumour cells proliferate within the lumen of the duct and are retained by a near-continuous myoepithelial cell layer, which lies in contact with the basement membrane (BM). Progression is marked by tumour cells breaching the myoepithelial cell-BM interface and invading into the surrounding stroma. Prior to the establishment of screening mammography, DCIS accounted for <2% of all diagnosed breast cancers, but through screening programmes DCIS now accounts for ~25% of all breast cancer diagnoses^2–4^. Despite the increased detection and treatment of DCIS, there has not been a concurrent decrease in invasive breast cancer (IBC) diagnosis^5^, suggesting that many cases of DCIS are overtreated that would not progress during a woman’s lifetime. Indeed, in several small series where DCIS was left untreated, owing to misdiagnosis, only ~40% progressed to invasive disease over 30 years^6–8^. Thus, there is an urgent clinical need to identify markers that will predict the progression of DCIS in order to better direct therapeutic intervention^9^.

Molecular approaches have been used extensively for decades to identify such markers that may predict DCIS progression, with most studies focusing on the comparison of tumour epithelial cells from DCIS with their invasive counterpart. Genomic profiling and gene expression analysis has not revealed any specific alterations associated with the progression to invasion^10–15^, and suggests DCIS exhibits a high level of similarity to IBC, supporting DCIS as a precursor to invasion^16^. More recent studies using advanced molecular approaches confirm this similarity, but also demonstrate through variations in clonal patterns that the mechanism of progression of DCIS is likely very diverse^17^. The lack of a clear ‘invasion signature’ led to a focus on the breast. microenvironment, comprising the myoepithelial, stromal and immune cells, implying key roles for them in the progression to invasive disease. In particular, the presence of a myoepithelial cell layer is characteristic of DCIS, and disruption to this interface is a hallmark of invasive progression. Myoepithelial cells play essential roles in mammary gland development and function, such that they maintain luminal epithelial cell polarity and induce ductal branching and differentiation during mammary gland development^18–21^. Studies have indicated that myoepithelial cells play a role in tumour suppression^19,22,23^ by secretion of protease inhibitors and downregulation of matrix metalloproteases (MMPs) that have inhibitory effects on tumour cell growth, invasion and angiogenesis^24–28^. In addition, tumour cells adjacent to a focally disrupted myoepithelial cell layer display gene expression changes associated with invasive properties, higher proliferation rates and associate with leukocyte infiltration^29–32^. Due to these tumour suppressive roles, myoepithelial cells are considered gatekeepers to tumour progression.

Whereas normal myoepithelial cells have been demonstrated to be tumour suppressive, several studies have identified that DCIS myoepithelial cells exhibit an altered phenotype, and are suggested to switch to a tumour promoter function^18,32–35^. We previously showed that DCIS myoepithelial cells exhibit *de novo* expression of integrin β6, which is predictive of DCIS progression to invasion and disease recurrence. Such that, integrin β6-positive DCIS cases recurred more rapidly than integrin β6-negative DCIS cases, at 2.3 years compared to 11.4 years, respectively^36^. In culture and in vivo studies identified that myoepithelial cell expression of integrin β6 enhanced breast tumour cell invasion through TGFβ-mediated upregulation of MMP9^36^. In support of our previous work, others have demonstrated, using in vitro studies, that tumour-associated myoepithelial cells secrete TGFβ to promote the invasive progression of DCIS cells due to enhancing epithelial-to-mesenchymal transition and basal-like phenotypes through activation of the TGFβ/SMAD signalling pathway^37,38^. However, the mechanisms inducing such alterations to DCIS myoepithelial cells are largely unknown.

Here, we show that mimicking the mechanical stretching of myoepithelial cells in DCIS duct expansion induces a DCIS myoepithelial cell phenotype associated with invasive progression. We demonstrate expression of myoepithelial cell integrin β6 and periductal fibronectin in DCIS associates with a higher propensity to progress to invasive breast cancer. Using primary and cell line models, we identify that integrin β6-positive myoepithelial cells promote the deposition of fibronectin. Together, myoepithelial cell integrin β6 and fibronectin promote the activation of TGFβ signalling to induce the secretion of BM-degrading proteases MMP9 and MMP13 that facilitate breast tumour cell invasion. Furthermore, we show that this DCIS myoepithelial cell phenotype is induced by mechanostimulation in a TGFβ-dependent manner. The findings indicate that mechanostimulation-mediated alterations to DCIS myoepithelial cell function contribute to invasion and that these biomarkers may be used to stratify patients for risk of DCIS progression.

## Results

### DCIS progression is accompanied by increased myoepithelial cell expression of integrin β6 and periductal fibronectin deposition

**Fig. 1:**
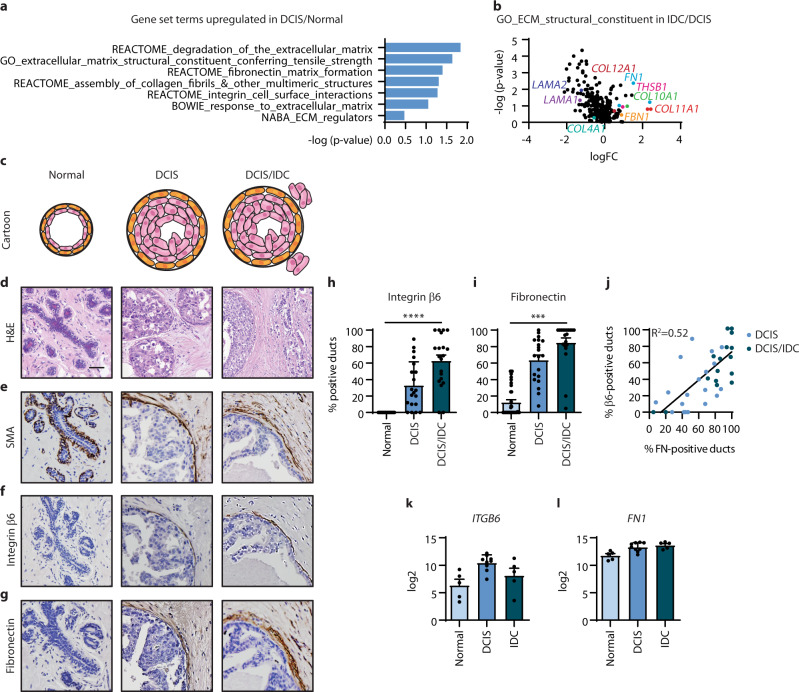
Myoepithelial expression of integrin β6 and periductal fibronectin deposition correlates with human DCIS progression. **a** GSEA of transcriptional profiles from GEO series GSE21422 showing upregulated extracellular matrix (ECM) remodelling and integrin interaction gene set terms associated DCIS (*n* = 9) compared to normal breast (*n* = 5). **b** Scatter plot of p-value (-log10) vs. log fold change (logFC) for gene expression from the GO_extracellular_matrix_constituent gene set for microarray data for IDC (*n* = 5) compared to DCIS (*n* = 9). **c** Cartoon depicting a normal duct, a DCIS duct without (pure DCIS) and with co-existent invasion (DCIS/IDC). **d** Haematoxylin and eosin (H&E) staining of human breast tissue samples featuring adjacent normal ducts, DCIS and DCIS/IDC. Scale bar, 100 μm. **e**–**g** Representative images of smooth-muscle actin (SMA) (**e**), integrin β6 (**f**) and fibronectin (**g**) by immunohistochemical staining in human breast tissue samples featuring adjacent normal ducts, DCIS and DCIS/IDC. **h**, **i** Bar graphs showing the mean and individual percentage of ducts positive for integrin β6 (**h**) or fibronectin (**i**) in adjacent normal (*n* = 40), DCIS (*n* = 20) and DCIS/IDC (*n* = 20) patient samples (error bars, +s.e.m). ****P < 0.0001 (h, ordinary one-way ANOVA and i, Kruskal–Wallis one-way ANOVA). See Supplementary Table 1 for patient information. **j**, Scatter plot depicting the linear regression of the percentage of positive DCIS ducts for integrin αvβ6 (β6) and fibronectin (FN) in serial human tissue sections as in (f,g) (*n* = 40). **k**, **l**, mRNA expression of *ITGB6* and *FN1* in normal breast tissues (*n* = 5), DCIS (*n* = 9) and IDC (*n* = 5) (error bars, +s.e.m). *P = 0.019 (**k**) and **P = 0.0023 (**l**) (ordinary one-way ANOVA).

Tissue fibrosis is a feature of breast cancers that associates with tumour progression. Fibrotic breast tumours display increased abundance of ECM proteins and remodelling enzymes and elevated integrin signalling^39–41^. We previously showed that myoepithelial cell expression of integrin β6 promotes the invasion of breast tumour cells through TGFβ-driven upregulation of the ECM remodelling enzyme, MMP9^36^. In a study comparing gene expression profiles in human breast tissue samples, DCIS showed a significant upregulation of ECM/integrin-related gene categories compared to normal breast (Fig. 1a)^42^. Plotting fold change versus p-values of the gene expression data illustrates several upregulated ECM proteins and downregulated BM proteins from the GO_Extracellular_Matrix_Structural_Constituent category in invasive ductal carcinoma (IDC) compared to DCIS (Fig. 1b). In particular, *FN1* was one of the most evident and consistent ECM alterations in IDC, which led us to speculate fibronectin as a driver of DCIS progression. To investigate whether a causal role exists between ECM remodelling, myoepithelial integrin signalling and DCIS progression, we first assessed the duct-by-duct expression of myoepithelial cell integrin β6 and periductal fibronectin in serial sections of DCIS tissues without (pure DCIS) and with (DCIS/IDC) invasive disease, as well as adjacent normal breast tissue (Fig. 1c, Supplementary Table 1). Haematoxylin and eosin (H&E) staining confirmed the presence of normal and DCIS ducts within these tissues, and the presence of invasion in DCIS/IDC tissues was used as a marker of DCIS progression (Fig. 1d). Normal and DCIS ducts show an intact myoepithelial cell layer, as shown by SMA immunohistochemistry (Fig. 1e). Immunohistochemical staining for myoepithelial integrin β6 revealed that the adjacent normal breast ducts had no expression, whereas DCIS ducts in all patients exhibited some expression of myoepithelial integrin β6 (Fig. 1f, h). The percent of positive DCIS ducts for myoepithelial integrin β6 was higher in high-grade pure DCIS (45%) compared with non-high grade pure DCIS (27%), and increased further in DCIS/IDC (68%), consistent with our previous reports that myoepithelial integrin β6 associates with DCIS progression to invasion (Fig. 1f, h and Supplementary Table 2)^36^. Immunohistochemical staining for periductal fibronectin revealed that the adjacent normal breast ducts had little expression (6%), whereas the percent of positive DCIS ducts was higher in DCIS/IDC (87%) compared with pure DCIS (68%), with no difference between non-high and high-grade pure DCIS (Fig. 1g, i and Supplementary Table 3). Matched duct scoring established a correlation between myoepithelial integrin β6 and periductal fibronectin expression (Fig. 1j and Supplementary Table 4). We also confirmed using the gene expression profile study of human breast tissue samples, a progressive increase in both *ITGB6* and *FN1* mRNA levels with DCIS progression to IDC (Fig. 1k, l). The data implicate a relationship between integrin β6-positive myoepithelial cells and fibronectin deposition surrounding the duct as a function of DCIS progression to invasion.

### Integrin β6-positive myoepithelial cells upregulate fibronectin expression

**Fig. 2:**
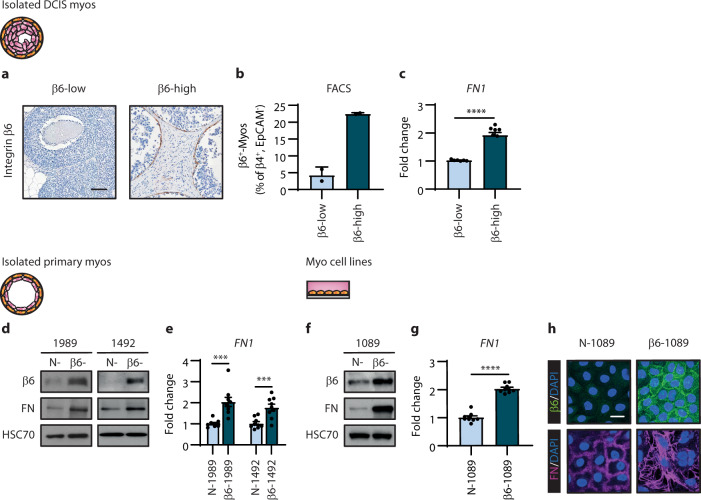
Integrin β6-positive myoepithelial cells upregulate fibronectin expression. **a** Representative images of integrin β6 by immunohistochemical staining in human DCIS tissue samples classified as low and high expression of integrin β6. Scale bar, 100 μm. **b** Bar graph showing the percent integrin β6-positive (β6^+^) myoepithelial (myo) cells (β4^+^EpCAM^-^) in human DCIS samples (*n* = 4) (error bars, +s.e.m). **c** Bar graphs showing qRT-PCR analysis for *FN1* using RNA isolated from DCIS myoepithelial cells with integrin β6-low (*n* = 2) and β6-high (*n* = 2) expression (error bars, +s.e.m). 3 technical replicates; ****P < 0.0001 (two-tailed *t*-test). **d** Immunoblots of integrin β6 and fibronectin (FN) in primary normal myoepithelial cells; 1989 and 1492, transfected with an empty vector (N-) or integrin β6 expression construct (β6-). HSC70 serves as loading control. Images are representative of three experiments. **e** Bar graphs showing qRT-PCR analysis for *FN1* using RNA isolated from primary normal myoepithelial cells, generated as in (**d**) (error bars, +s.e.m). *n* = 3 biological replicates, 3 technical replicates; 1989 ***P = 0.0003 and ***P = 0.0009 (two-tailed *t*-test). **f** Immunoblots of integrin β6 and fibronectin in myoepithelial cell lines; N-1089 and β6-1089. HSC70 serves as loading control. Images are representative of three experiments. **g** Bar graphs showing qRT-PCR analysis for *FN1* using RNA isolated from myoepithelial cell lines as in (**f**). *n* = 3 biological replicates, 3 technical replicates; ****P < 0.0001 (two-tailed *t*-test). **h** Representative images of integrin β6 (green) and fibronectin (FN, magenta) by immunofluorescent staining in myoepithelial cell lines as in (**f**). Nuclei were counterstained with DAPI (blue). Scale bar, 20 μm. Images are representative of three experiments.

We next investigated the potential relationship between myoepithelial integrin β6 and fibronectin deposition. For this purpose, we analysed freshly isolated primary DCIS and normal myoepithelial cells, as well as established myoepithelial cell lines, without and with the expression of integrin β6^36^. To select appropriate DCIS samples for further analysis, we first assessed the expression of integrin β6 using immunohistochemical staining of DCIS breast tissues with patient-matched DCIS ductal organoid preparations available (Fig. 2a). Two integrin β6-low and two -high DCIS ductal organoid samples were then selected. FACS analysis of these samples confirmed there was an increased frequency of integrin β6-positive myoepithelial cells in integrin β6-high DCIS compared to integrin β6-low DCIS (Fig. 2b). Quantitative reverse transcriptase-PCR (qRT-PCR) analysis revealed higher levels of *FN1* in myoepithelial cells in integrin β6-high DCIS compared to integrin β6-low DCIS (Fig. 2c). Furthermore, induction of integrin β6 expression in isolated primary normal myoepithelial cells (β6-1989 and β6-1492) revealed an increase in fibronectin compared to the control cell populations (N-1989 and N-1492; Fig. 2d, e). Similarly, using established myoepithelial cell lines; we identified higher levels of fibronectin in the integrin β6-positive myoepithelial cell line (β6-1089) compared to the integrin β6-negative myoepithelial cell line (N-1089; Fig. 2f–h). These findings support that integrin β6-positive myoepithelial cells stimulate the deposition of fibronectin into the periductal microenvironment.

### Tumour promoting phenotype of integrin β6-positive myoepithelial cells is enhanced by fibronectin-mediated activation of TGFβ signalling

**Fig. 3:**
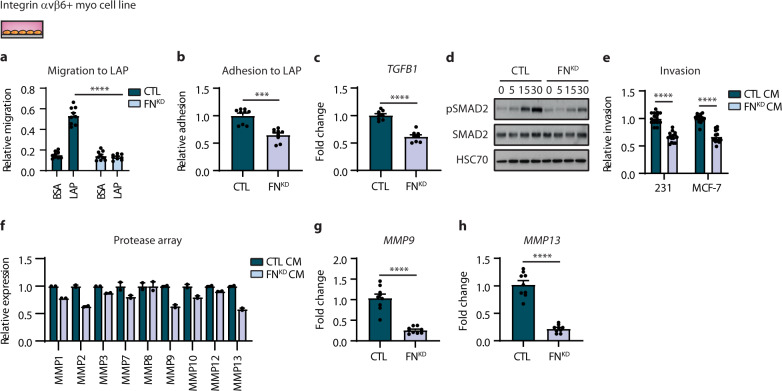
Integrin β6-positive myoepithelial cells promote breast cancer cell invasion by fibronectin-mediated activation of TGFβ signalling. **a** Migration assay for myoepithelial cell line; β6-1089 transfected with a control (CTL) or fibronectin-targeted (FN^KD^) siRNA, to BSA and LAP (error bars, +s.e.m). *n* = 3 biological replicates, 3 technical replicates; ****P < 0.0001 (two-tailed *t*-test). **b** Adhesion assay for myoepithelial cell lines generated as in (**a**) to LAP (error bars, +s.e.m). *n* = 3 biological replicates, 3 technical replicates; ***P = 0.0001 (two-tailed *t*-test). **c** Bar graph showing qRT-PCR analysis for *TGFB1* using RNA isolated from myoepithelial cell lines generated as in (**a**). *n* = 3 biological replicates, 3 technical replicates; ****P < 0.0001 (two-tailed *t*-test). **d** Immunoblots of phospho-SMAD2 (pSMAD2) and SMAD2 in myoepithelial cell lines generated as in (**a**), and stimulated with 5 ng/mL TGFβ (time in min). HSC70 serves as loading control. Images are representative of three experiments. **e** Invasion assay for MDA-MB-231 (231) and MCF-7 cells using conditioned media (CM) isolated from myoepithelial cell lines generated as in (**a**) (error bars, +s.e.m). *n* = 3 biological replicates, 4-6 technical replicates; ****P < 0.0001 (two-tailed *t*-test). **f** Array analysis for pro*t*eases using conditioned media isolated from myoepithelial cell lines generated as in (**a**) (error bars, +s.e.m). *n* = 1 biological replicate, 2 technical replicates. **g**, **h** Bar graphs showing qRT-PCR analysis for *MMP9* (**g**) and *MMP13* (**h**) using RNA isolated from myoepithelial cell lines generated as in (**a**) (error bars, +s.e.m). *n* = 3 biological replicates, 3 technical replicates; ****P < 0.0001 (two-tailed *t*-test).

Myoepithelial integrin β6 promotes the invasion of breast tumour cells in a TGFβ-mediated manner, and integrin β6 activation of TGFβ requires a mechanically resistant fibronectin matrix^36,43^. This raises the possibility that integrin β6-positive myoepithelial cells promote breast tumour cell invasion by stimulating TGFβ in a fibronectin-dependent manner. We therefore examined whether fibronectin could modulate the activation of TGFβ in integrin β6-positive myoepithelial cells, and if this elevated breast tumour cell invasion. We observed a decrease in integrin β6-positive myoepithelial cell line (β6-1089) migration and adhesion to latency-associated peptide (LAP) with fibronectin knockdown (FN^KD^) compared to control (CTL) (Fig. 3a, b). qRT-PCR analysis revealed reduced levels of *TGFB1* in integrin β6-positive myoepithelial cell line with fibronectin knockdown compared to control (Fig. 3c). Immunoblots revealed that the knockdown of fibronectin in integrin β6-positive myoepithelial cell line reduced the ability of TGFβ to stimulate phosphorylation of SMAD2 compared to control (Fig. 3d). These results suggest that fibronectin expression by integrin β6-positive myoepithelial cells enhances its TGFβ binding and activating properties.

We next explored whether fibronectin could enhance the tumour-promoting function of integrin β6-positive myoepithelial cells. Invasion assays revealed that the knockdown of fibronectin in the integrin β6-positive myoepithelial cell line led to a decrease in breast tumour cell invasion in vitro compared to control (Fig. 3e). Consistently, knockdown of fibronectin in the integrin β6-positive myoepithelial cell line revealed the broad downregulation in protease expression, with the most differentially expressed proteases implicated in promoting breast cancer invasion through degradation of the BM, including MMP9 and MMP13 (Fig. 3f). qRT-PCR analysis confirmed reduced levels of *MMP9* and *MMP13* in integrin β6-positive myoepithelial cell line following fibronectin knockdown compared to control (Fig. 3g, h). These findings suggest that DCIS myoepithelial cells exhibit a tumour-promoting phenotype mediated by both integrin β6 and fibronectin.

### DCIS myoepithelial cells enhance progression to invasion by increasing the expression of MMP13

**Fig. 4:**
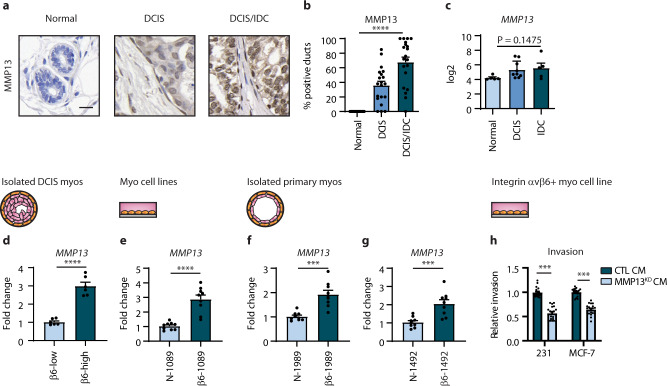
MMP13 expression correlates with human DCIS progression to invasion and promotes breast cancer cell invasion in vitro. **a** Representative images of MMP13 by immunohistochemical staining in human breast tissue samples featuring adjacent normal ducts, DCIS and DCIS/IDC. Scale bar, 20 μm. **b** Bar graphs showing the mean and individual percentage of ducts positive for MMP13 in adjacent normal (*n* = 40), DCIS (*n* = 20) and DCIS/IDC (*n* = 20) patient samples (error bars, +s.e.m). ****P < 0.0001 (Kruskal–Wallis one-way ANOVA). **c** mRNA expression of *MMP13* in normal breast tissues (*n* = 5), DCIS (*n* = 9) and IDC (*n* = 5) (error bars, +s.e.m). * *P* = 0.0373 (Kruskal–Wallis one-way ANOVA). **d–g** Bar graphs showing qRT-PCR analysis for *MMP13* using RNA isolated from DCIS myoepithelial cells with integrin β6-low and β6-high expression (**d**), myoepithelial cell lines; N-1089 and β6-1089 (**e**) and from primary normal myoepithelial cells; 1989 (**f**) and 1492 (**g**) transfected with an empty vector (N-) or integrin β6 expression construct (β6-) (error bars, +s.e.m). *n* = 3 biological replicates, 3 technical replicates; ****P < 0.0001 (d,e), ***P = 0.0001 (**f**) and ***P = 0.0006 (**g**) (two-tailed *t*-test). **h** Invasion assay for MDA-MB-231 (231) and MCF-7 cells using conditioned media (CM) isolated from myoepithelial cell line; β6-1089 transfected with a control (CTL) or MMP13-targeted (MMP13^KD^) siRNA (error bars, +s.e.m). *n* = 3 biological replicates, 4-6 technical replicates; ***P = 0.0001 (two-tailed *t*-test).

MMP13 expression by stromal cells adjacent to DCIS has been implicated in promoting its progression to invasion^44^. We next examined whether myoepithelial MMP13 expression could promote the progression of DCIS to invasion. We first analysed our cohort of DCIS tissue samples by immunohistochemical staining for MMP13. This revealed that the adjacent normal breast tissue had no expression, whereas the percentage of positive DCIS ducts was higher in DCIS/IDC (72%) compared with pure DCIS (40%), with no difference between non-high and high grade pure DCIS (Fig. 4a, b and Supplementary Table 5). We also confirmed, using the gene expression profile study of human breast tissue samples, a progressive increase in *MMP13* mRNA levels with the progression of DCIS (Fig. 4c). Consistently, qRT-PCR analysis revealed that levels of *MMP13* were increased in all integrin β6-positive primary and cell line models of myoepithelial cells examined, compared to their integrin β6-negative counterparts (Fig. 4d–g). Furthermore, invasion assays revealed that the knockdown of MMP13 in integrin β6-positive myoepithelial cell line led to a decrease in breast tumour cell invasion in vitro compared to control (Fig. 4h). These findings identify MMP13 as a key protease elevated in DCIS progression, and implicate the integrin β6-fibronectin-MMP13 axis in the pro-tumourigenic properties of DCIS myoepithelial cells.

### Mechanical stretching of normal myoepithelial cells induces a DCIS phenotype associated with integrin β6 expression

**Fig. 5:**
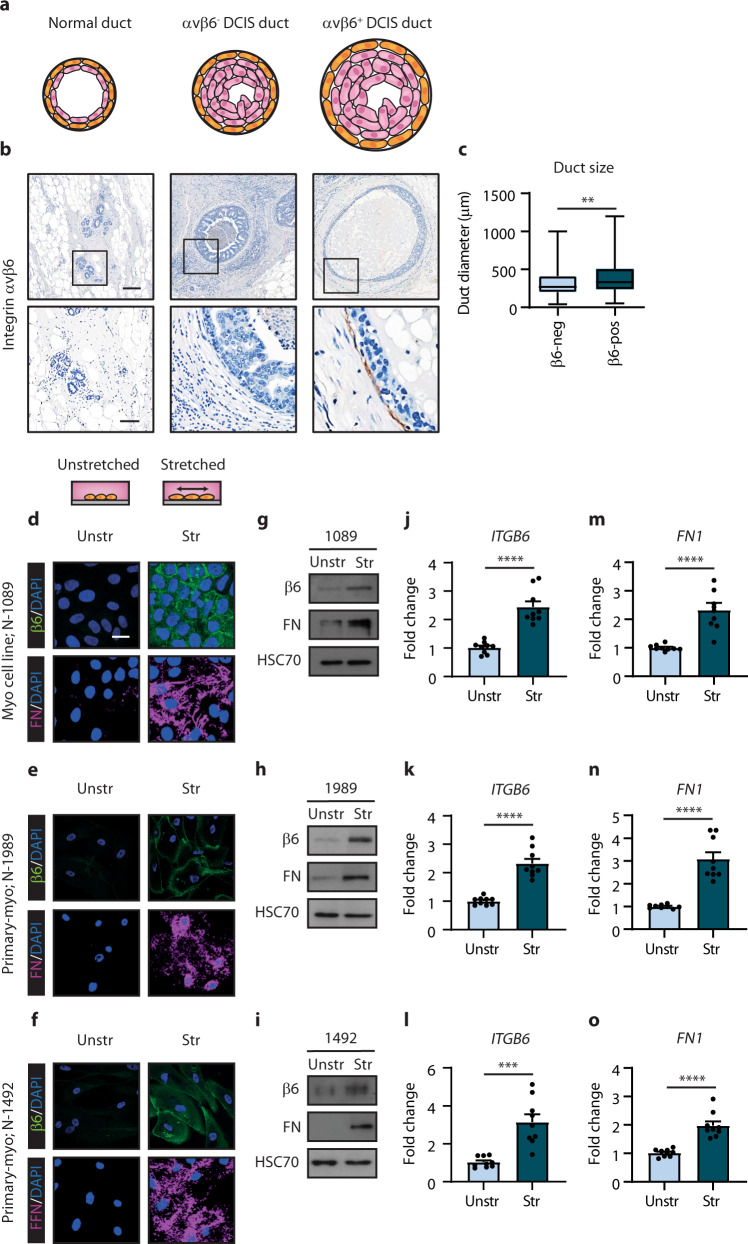
Mechanical stretching normal myoepithelial cells induces integrin β6 expression. **a** Cartoon depicting duct sizes for a normal duct, and a DCIS duct without and with integrin β6 expression (not to scale). **b** Representative images of integrin β6 by immunohistochemical staining in human breast tissue samples featuring adjacent normal ducts (40 μm), and an integrin β6-negative and β6–positive DCIS duct. Scale bar, (top) 200 μm and (bottom) 100 μm. **c** Box and whiskers plot showing DCIS duct sizes with (*n* = 656) or without (*n* = 713) integrin β6 expression. Box plot represents the median (central line) and interquartile range (IQR; box), and whiskers represent the maximum and minimum. **P = 0.0312 (two-tailed *t*-test). **d–f** Representative images of integrin β6 (green) and fibronectin (FN, magenta) by immunofluorescent staining in unstretched (Unstr) or stretched (Str) myoepithelial cell line; N-1089 (**d**) and primary normal myoepithelial cells; N-1989 (**e**) and N-1492 (**f**). Nuclei were counterstained with DAPI (blue). Scale bar, 20 μm. Images are representative of three experiments. **g**–**i** Immunoblots of integrin β6 and fibronectin (FN) in unstretched or stretched myoepithelial cells generated as in (**d–f**). HSC70 serves as loading control. Images are representative of three experiments. **j–l** Bar graphs showing qRT-PCR analysis for *ITGB6* using RNA isolated from unstretched or stretched myoepithelial cell line; N-1089 (**j**) and primary normal myoepithelial cells; N-1989 (**k**) and N-1492 (**l**) (error bars, +s.e.m). *n* = 3 biological replicates, 3 technical replicates; ****P < 0.0001 (**j**, **k**) and ***P = 0.0001 (**l**) (two-tailed *t*-test). **m–o**, Bar graphs showing qRT-PCR analysis for *FN1* using RNA isolated from unstretched or stretched myoepithelial cells generated as in (**j–l**) (error bars, +s.e.m). *n* = 3 biological replicates, 3 technical replicates; ****P < 0.0001 (two-tailed *t*-test).

We next investigated potential mechanisms whereby a DCIS myoepithelial cell phenotype could be induced. DCIS is characterised by the proliferation of tumour cells within the duct, which results in the expansion of the duct and as a consequence, stretching of the myoepithelial cell layer (Fig. 5a, Supplementary Fig. 1a–e). We assessed DCIS duct size and identified that integrin β6-positive DCIS ducts (460 μm) on average were larger than integrin β6-negative DCIS ducts (380 μm) (Fig. 5b, c, Supplementary Table 6). To mimic the stretching of the myoepithelial cell layer as seen in DCIS expansion, we applied a 10% static, equibiaxial stretch to isolated primary and cell line models of normal myoepithelial cells. Consistent with our tissue study, mechanical stretching of the normal myoepithelial cell line (N-1089) led to an increase in the expression of integrin β6 and fibronectin (Fig. 5d, g, j, m). Isolated primary normal myoepithelial cells (N-1989 and N-1492) exposed to mechanical stretching similarly showed a significant increase in integrin β6 and fibronectin expression (Fig. 5e, h, k, n and f, i, l, o, respectively). These data suggest that the DCIS myoepithelial cell phenotype observed here may be regulated, at least in part, by evolving mechanics in the development of DCIS.

**Fig. 6:**
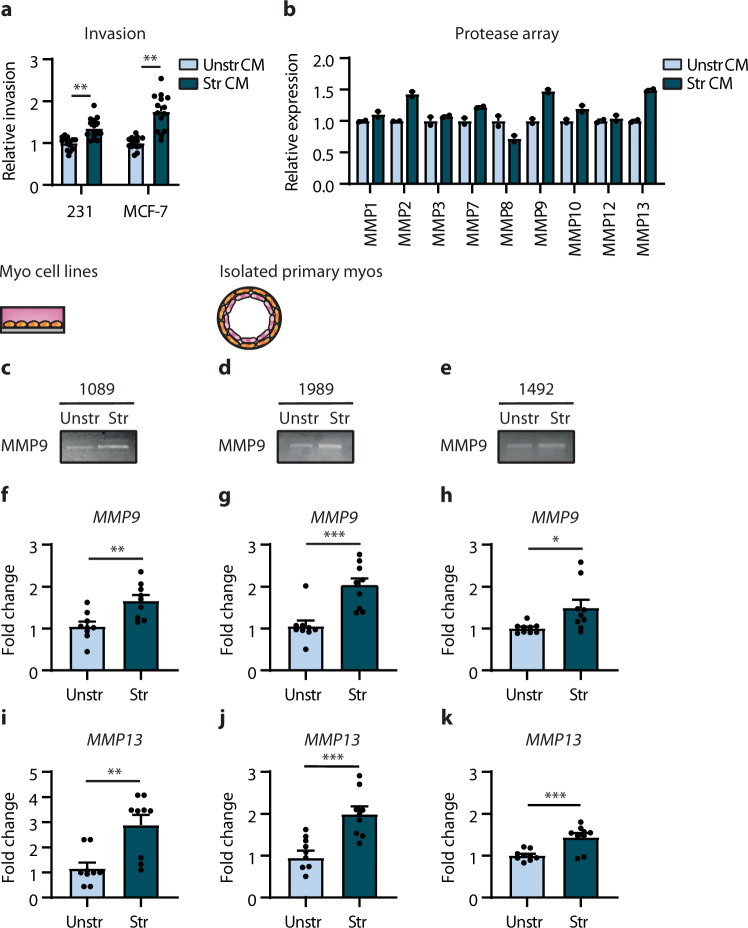
Mechanical stretching normal myoepithelial cells activates a tumour promoting phenotype. **a** Invasion assay for MDA-MB-231 (231) and MCF-7 cells using conditioned media (CM) isolated from unstretched (Unstr) or stretched (Str) normal myoepithelial cell line; N-1089 (error bars, +s.e.m). *n* = 3 biological replicates, 5 technical replicates; ****P < 0.0001 (two-tailed *t*-test). **b** Array analysis for proteases using conditioned media isolated from unstretched or stretched myoepithelial cell line; N-1089 (error bars, +s.e.m). *n* = 1 biological replicate, 2 technical replicates. **c**–**e** Gelatin zymography for MMP9 activity using conditioned media isolated from unstretched or stretched myoepithelial cell line; N-1089 (**c**) and primary normal myoepithelial cells; N-1989 (**d**) and N-1492 (**e**). Images are representative of three experiments. **f–h** Bar graphs showing qRT-PCR analysis for *MMP9* using RNA isolated from unstretched or stretched myoepithelial cell line; N-1089 (**f**) and primary normal myoepithelial cells; N-1989 (**g**) and N-1492 (**h**) (error bars, +s.e.m). *n* = 3 biological replicates, 3 technical replicates; **P = 0.0035 (**f**), ***P = 0.0005 (**g**) and *P = 0.0232 (**h**) (two-tailed *t*-test). **i–k** Bar graphs showing qRT-PCR analysis for MMP13 using RNA isolated from unstretched or stretched myoepithelial cell line; N-1089 (**i**) and primary normal myoepithelial cells; N-1989 (**j**) and N-1492 (**k**) (error bars, +s.e.m). *n* = 3 biological replicates, 3 technical replicates; **P = 0.0017 (**i**), ***P = 0.0007 (**j**) and ***P = 0.0009 (**k**) (two-tailed *t*-test).

We next explored whether mechanical stretching could induce the associated tumour-promoting phenotype seen in DCIS myoepithelial cells. Invasion assays revealed both normal primary and cell line models of myoepithelial cells exposed to stretch-enhanced breast tumour cell invasion in vitro, compared to the unstretched controls (Fig. 6a, Supplementary Fig. 2a, b). Consistently, mechanical stretching of all myoepithelial cells revealed a broad upregulation in protease expression compared to unstretched controls (Fig. 6b; Supplementary Fig. 2c, d). Indeed, stretched normal primary and cell line models showed an increase in MMP9 and MMP13 (Fig. 6b; Supplementary Fig. 2c, d). Gelatin zymography confirmed elevated MMP9 activity in all of the stretched primary and cell line models of normal myoepithelial cells examined, compared to the unstretched controls (Fig. 6c–e). Furthermore, qRT-PCR analysis confirmed enhanced levels of *MMP9* and *MMP13* in all normal myoepithelial cells exposed to stretch compared to unstretched controls (Fig. 6f–k). These results show an association between duct expansion and induction of myoepithelial integrin β6 expression, and functionally link myoepithelial cell stretch to generation of a pro-tumourigenic phenotype. These data suggests that evolving tissue mechanics during DCIS development could induce the tumour-promoting phenotype of DCIS myoepithelial cells.

### DCIS myoepithelial phenotype induced by mechanical stretching is mediated by TGFβ signalling

**Fig. 7:**
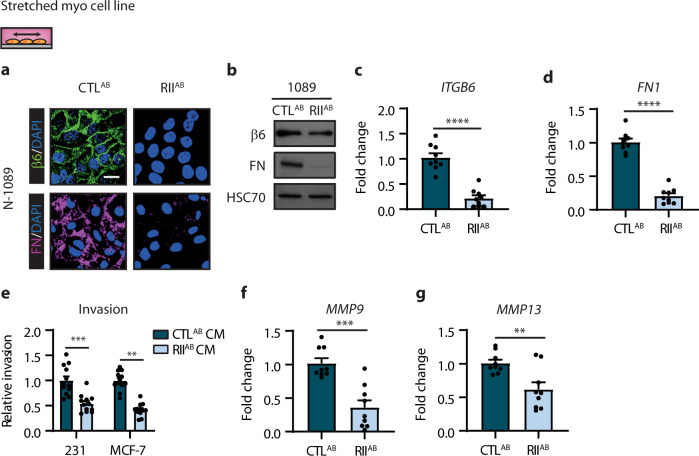
TGFβ signalling regulates a mechanically induced tumour promoting phenotype in normal myoepithelial cells. **a** Representative images of integrin β6 (green) and fibronectin (FN, magenta) by immunofluorescent staining in stretched normal myoepithelial cell line; N-1089 treated with a control (CTL^AB^) or TGFβRII (RII^AB^) blocking antibody. Nuclei were counterstained with DAPI (blue). Scale bar, 20 μm. **b** Immunoblots of integrin β6 and fibronectin in stretched normal myoepithelial cell lines generated as in (**a**). HSC70 serves as loading control. Images are representative of three experiments. **c**, **d** Bar graphs showing qRT-PCR analysis for *ITGB6* (**c**) and *FN1* (**d**) using RNA isolated from stretched normal myoepithelial cell lines generated as in (**a**) (error bars, +s.e.m). *n* = 3 biological replicates, repeated 3 times; ****P < 0.0001 (two-tailed *t*-test). **e** Invasion assay for MDA-MB-231 (231) and MCF-7 cells using conditioned media (CM) isolated from stretched normal myoepithelial cell lines generated as in (**a**) (error bars, +s.e.m). *n* = 3 biological replicates, repeated 4 times; MDA-MB-231 ***P = 0.0001 and MCF-7 ****P < 0.0001 (two-tailed *t*-test). **f**, **g** Bar graphs showing qRT-PCR analysis for *MMP9* (**f**) and *MMP13* (**g**) using RNA isolated from stretched normal myoepithelial cell lines generated as in (**a**) (error bars, +s.e.m). *n* = 3 biological replicates, repeated 3 times; ***P = 0.0001 (**f**) and **P = 0.0041 (two-tailed *t*-test).

We next examined the relationship between mechanostimulation, TGFβ signalling and induction of the DCIS myoepithelial cell phenotype. Inhibition of the TGFβRII (RII^AB^) with a blocking antibody in stretched normal myoepithelial cell line (N-1089) abrogated the upregulation of integrin β6 and fibronectin compared to control (CTL^AB^) (Fig. 7a-d). Invasion assays revealed inhibition of the TGFβRII reduced breast tumour cell invasion in vitro, compared to control (Fig. 7e). Consistently, qRT-PCR analysis showed reduced *MMP9* and *MMP13* levels in these cells (Fig. 7f, g). These data suggest mechanical stimulation of TGFβ signalling could be essential in promoting, further, the DCIS myoepithelial phenotype associated with progression to invasion, and that these markers may be used to identify patients at higher risk for invasive progression.

## Discussion

Here we demonstrate that physical cues induce a tumour-promoting phenotype in myoepithelial cells. Our findings support the critical role played by myoepithelial cells in DCIS progression and are consistent with our prior data implicating myoepithelial integrin β6 in driving invasion^36^. We elaborate upon these prior studies by identifying a molecular mechanism whereby tissue mechanics increases myoepithelial integrin β6 and periductal fibronectin expression through the tension-dependent enhancement of TGFβ signalling that promotes BM-degrading proteases that facilitate invasive progression. Accordingly, our study identifies phenotypic changes in myoepithelial cells that may stratify women who are at higher risk of invasive progression and are therefore ideal candidates for more aggressive therapies. Our findings also suggest that treatments aimed at inhibiting DCIS myoepithelial cell function would constitute novel treatment modalities and could potentially be used to lower the number of women undergoing invasive surgery.

Previous work examining DCIS progression has attributed the transition to invasion to specific features of the breast microenvironment. Such prior studies have identified genes differentially expressed between DCIS and invasion that encode for cell adhesion and ECM-related proteins^15,42^. In support of this, recent high-dimensional analysis using multiplex ion beam imaging by time of flight (MIBI-TOF) to examine the histological stages of DCIS progression identified the most distinctive property delineating DCIS from invasive disease was an increase in stromal fibrosis associated with collagen deposition and remodelling, and cancer-associated fibroblast frequency and proliferation^45^. This study further identified that DCIS recurrence risk is heavily influenced by myoepithelial morphology and phenotype^45^. Here, we quantified higher myoepithelial integrin β6 and periductal fibronectin expression in DCIS ducts associated with invasive disease in tissues, supporting the role for perturbed integrin signalling, ECM remodelling and an altered myoepithelial cell phenotype in driving DCIS progression. Interestingly, these observed changes in integrin β6 and fibronectin were more frequently observed in high-grade DCIS ducts than in non-high grade DCIS ducts, which could explain their progression to invasion more quickly, although all grades have equal potential to progress^8^. Matched duct analysis in tissues implicated a relationship between myoepithelial integrin β6 and periductal fibronectin expression. Consistently, our analysis of isolated primary DCIS and normal myoepithelial cells, as well as established myoepithelial cell lines, corroborates our clinical findings, revealing significantly increased fibronectin expression by integrin β6-positive myoepithelial cells. Together these data suggest that DCIS myoepithelial cells exhibit an ECM remodelling phenotype. Using our myoepithelial cell line models, we implicate a functional relationship between myoepithelial integrin β6 and fibronectin in driving breast tumour cell invasion in vitro, through TGFβ-mediated protease activity. Our studies support the finding that MMP13 is strongly associated with DCIS progression^44^, thereby linking this pathway to patient outcome.

Our tissue analysis identified an increased DCIS duct size associated with myoepithelial integrin β6 positivity. We hypothesized that physical cues in DCIS due to proliferative expansion of ducts may trigger these phenotypic and functional changes to myoepithelial cells. Indeed, by modelling the physical tension seen in myoepithelial cells during DCIS expansion, we identified a consistent and significant upregulation of integrin β6 and fibronectin. These changes were associated with the induction of our observed tumour-promoting phenotype- upregulation of MMP9 and MMP13. Furthermore, we identified a tension-dependent requirement of TGFβ signalling that facilitates the induction of this phenotype in normal myoepithelial cells. These findings imply that physical cues in DCIS regulate myoepithelial cell phenotype and function that critically regulates progression to invasion. In support of mechanical regulation of myoepithelial cells, we have previously shown that increasing substrate rigidity, as seen in DCIS progression, resulted in the loss of homeostasis force generation by integrin β6-positive myoepithelial cells, which could alter their ability to function as a barrier to invasive dissemination into the stroma^46^. Indeed, studies have shown that myoepithelial cells act as a dynamic barrier to tumour cell invasion that relies upon their contractility and adhesion^47^. We suggest that this function is compromised in integrin β6-positive myoepithelial cells, that along with their ability to degrade the BM, facilitates the breaching of tumour cells into the surrounding stroma. Our studies highlight the importance of future studies utilizing mechanical models to investigate mechanisms by which the myoepithelial cell barrier is lost. In summary, we show here that tension to myoepithelial cells induces integrin β6 and fibronectin expression, which in turn activates TGFβ signalling to stimulate the expression of BM-degrading MMPs that promote invasion of tumour cells into the stroma. Our findings thus provide new evidence supporting a mechanism regulating DCIS myoepithelial cell phenotype that facilitates invasive progression, and identifies biomarkers that may be used to stratify women with DCIS.

## Methods

### Gene expression microarray analysis

Array dataset from GEO (Gene Expression Omnibus) database with the accession ID #GSE21422 was selected to assess expression profile changes in an independent cohort of human breast tissues. The samples consist of normal healthy breast (*n* = 5), DCIS (*n* = 9) and IDC (*n* = 5) tissues. Gene ontology was performed using Gage (version 2.36.0) with gene lists from MsigDB version 7.2.

### Human breast tissue collection and processing

Tissue specimens were donated by women undergoing surgery for DCIS, and tissues were collected as formalin-fixed and paraffin-embedded (FFPE) or as fresh tissue for FACS-mediated DCIS myoepithelial cell isolation. Samples of DCIS without evidence of invasion (pure DCIS; *n* = 20) and DCIS with co-existent invasion (DCIS/IDC; *n* = 20) were selected for immunohistochemical analyses. DCIS was classified as low, intermediate, and high grade based on nuclear characteristics, and grouped as non-high grade (low and intermediate) and high grade for analysis. Clinicopathologic details of tissues analysed by immunohistochemistry are provided in Supplementary Table 1. Tissue specimens were donated by women undergoing reduction mammoplasty, and tissues were collected as fresh tissue for FACS-mediated human normal myoepithelial cell isolation and subsequent cell culture. All human breast tissues were collected from consenting patients undergoing surgery at Barts Health NHS Trust London between 2000 and 2015. Samples were stored and analysed with deidentified labels to protect patient data in accordance with data under the terms of the Barts Cancer Institute Breast Tissue Bank (REC no: 15/EE/0192).

### Immunohistochemical staining

Tissue sections were dewaxed in xylene, rehydrated in graded alcohols, endogenous peroxidases were blocked with 3% H_2_O_2_ in methanol, antigen retrieved in pepsin solution (Life Technologies) and blocked in 5% BSA in PBS, followed by incubation with primary antibodies specific to SMA (Dako, 1A4, 1:500), integrin β6 (Calbiochem, 442.5C4, 1:800), fibronectin (Sigma, IST-4, 1:400), MMP13 (abcam, VIIIA2, 1:100) or p63 (abcam, 4A4, 1:50) overnight at 4 °C. Sections were washed with PBS prior to incubation with anti-mouse biotinylated F(ab’)2, developed using ABC reagent and DAB (Vector Laboratories), counterstained with haematoxylin, dehydrated in graded alcohols and mounted with DPX.

### Immunohistochemical analysis

Stained sections were imaged using a 3DHISTECH Panoramic digital slide scanner and analysed using QuPath (version 0.2.3) open-source software^48^. Immunohistochemical analysis was performed on a duct-by-duct basis. Ducts were numbered and identified as either; normal, benign or DCIS within each case, by an expert breast pathologist. For expression analysis; each duct was then scored as negative or positive for integrin β6, fibronectin and MMP13 in serial sections. For samples stained with fibronectin, periductal staining was measured, which was defined as a 50 µm region bordering DCIS lesions. For DCIS duct size analysis, only cross-sectional ducts were included. For cell and nuclear size analysis; myoepithelial cells positive for SMA or p63 were segmented by semi-automated detection, and cell and nuclear morphology features were extracted.

### Human DCIS and normal myoepithelial cell isolation

Fresh breast tissues were digested into ductal organoids by manual chopping followed by digestion with 5% FBS in RPMI containing 1 mg/ml collagenase 1 A (Roche Life Science) and 1 mg/ml hyaluronidase (Sigma), overnight at 37 °C. Ductal organoids were then digested to a single-cell suspension with 0.05%/0.02% trypsin/EDTA solution (Hyclone) containing 0.4 mg/mL DNase (Roche Life Science) for 15 minutes at 37 °C for subsequent cell isolation. Normal and DCIS myoepithelial cells were isolated using fluorescence-activated cell sorting (FACS). Cells were incubated for 45 min at 4 °C with the following human-specific primary antibodies at 1 μg/1 million cells: for normal cell suspensions EpCAM-FITC (BD Biosciences, EBA-1) and CD10-APC, (BD Biosciences, HI10a) and for DCIS cell suspensions; EpCAM-PE (BD Biosciences, EBA-1), integrin β4-Alexa-Fluor 488 (Invitrogen, 422325) and integrin β6-APC (R&D Systems, 437211). Cells were then incubated with 4′,6-diamidino-2-phenylindole (DAPI) for 4 °C for 10 min to distinguish live/dead cells. EpCAM^+^ cells were gated out to avoid epithelial contamination, and CD10^+^ normal myoepithelial cells or integrin β4 + /β6 + DCIS myoepithelial cells were sorted into RPMI (Sigma) with 10% foetal bovine serum (FBS, Sigma). BD FACSAria II cell sorters were used to conduct cell sorting using FACSDiva software (BD Biosciences). Data were analysed using FlowJo software (Tree Star). Isolated myoepithelial cells were collected and used for primary cell culture or RNA isolation.

### Primary and cell line culture

All cell lines were tested for mycoplasma contamination by PCR-based method and confirmed negative for mycoplasma before experiments and were maintained at 37 °C in a humidified 5% CO_2_ atmosphere. Isolated normal myoepithelial cells were cultured in HuMEC Ready Medium (Thermo Fisher Scientific) supplemented with 50 μg/mL bovine pituitary extract (BPE, Invitrogen), 0.5 μg/mL hydrocortisone, 10 ng/mL EGF, 5 μg/mL insulin, 0.5 μg/mL fungizone (Invitrogen) and 10 μg/mL gentamicin (Sigma) and cultured on plates coated with 10 µg/cm^2^ type I collagen (Corning). Myoepithelial cell lines; N-1089 and β6-1089 were grown in Nutrient Mixture Ham’s F-12 (Sigma) supplemented with 10% FBS, 1 μg/mL hydrocortisone (Sigma), 10 ng/mL Epidermal Growth Factor (EGF, Sigma), and 10 μg/mL insulin (Sigma). Breast cancer cell lines; MDA-MB-231 and MCF-7 were obtained from American Type Culture Collection (ATCC), verified with STR profiling (LGC Standards, Teddington, UK, tracking number 710081047), and grown in DMEM (Sigma) supplemented with 10% FBS.

### DNA and siRNA transfection

Cells were reverse transfected with 10 μg integrin β6 pcDNA3.1 neo, a gift from Dean Sheppard (Addgene, plasmid 13580), or pcDNA3.1 empty vector (Invitrogen) using the jetPRIME transfection reagent (PolyPlus). Cells were reverse transfected with 9 nM fibronectin, MMP13 or non-targeting control (CTL) siRNA (Dharmacon) using interferin transfection reagent (Polyplus). Functional assays were carried out 48 hr post-transfection.

### TGFβ stimulation

Cells were cultured in serum-free media for 24 h prior to stimulation. Cells were then washed in PBS to remove residual media and were then stimulated with 5 ng/mL recombinant human active TGFβ1 (PeproTech) in serum-free media for 5, 15 and 30 min.

### TGFβRII inhibition

Cells were incubated with 10 μg/mL TGFβRII-blocking antibody (R&D Systems) or IgG isotype control antibody (Merck Millipore, GC270) in serum-free media for 20 min at 4 °C on a rotating-wheel before plating. Functional assays were carried out 48 h postantibody treatment.

### Conditioned media

Conditioned media (CM) was generated by culturing cells in serum-free media for 48 h. CM was concentrated 24-fold with centrifugal filters (Fisher) with 3 K molecular weight cut off (MWCO) at 4000 g for 45 min at 4 °C.

### Transwell® migration and invasion assays

Motility assays were performed using Transwell® migration inserts (8 μm pore size, polycarbonate membrane, Corning). For migration assays the underside of inserts were coated with 0.5 μg/mL recombinant human latency-associated peptide of TGFβ1 (LAP, R&D Systems) or 0.1% BSA; for invasion assays the top of each insert was coated with Matrigel (BD Biosciences) diluted 1:3 in DMEM. Migrating and invading MDA-MB-231 and MCF-7 cells to the lower chamber were counted after 8 h, and 24 or 48 h incubation, respectively, using a CASY counter (Schärfe System). For migration, total cell count for each sample was calculated by adding the counts of the upper and lower chambers. Relative cell migration was then calculated by using the lower chamber count versus total cell count. For invasion, for each sample only the lower chamber was counted. Relative cell invasion was then calculated by normalising to the control.

### Adhesion Assay

96-well plates were coated with 0.5 μg/mL recombinant human LAP (R&D Systems) or 0.1% BSA and incubated for 1 h at 37 °C. Cells were then seeded and allowed to adhere for 1 hr at 37 °C before fixing in methanol and staining with 0.1% crystal violet. Stained cells were dissolved with 30% acetic acid and absorbance read at 550 nm. Background binding to BSA was subtracted from LAP, and relative adhesion was calculated by normalising to the control.

### Immunoblotting

Total cellular protein was isolated using RIPA buffer (50 mM Tris-HCl pH 7.4, 150 mM NaCl, 1% IPEGAL CA-630, 0.1% Na-DOC, 1 mM EDTA) supplemented with protease and phosphatase inhibitor cocktails (EMD Millipore). Lysates containing equal amounts of protein (30 μg) were electrophoresed in 6-10% SDS-PAGE gel, electroblotted to nitrocellulose membrane (Amersham). Membranes were then blocked with 0.1% Tween-20 in TBS (TBS-T) supplemented with 5% milk for 1 hr prior to incubation with primary antibodies specific for integrin β6 (Santa Cruz, C-19, 1:500), fibronectin (FN, Sigma, IST-4, 1:500), phospho-SMAD2 S465/467 (CST, 138D4, 1:500), SMAD2 (CST, 86F7, 1:500) and HSC70 (Santa Cruz, B-6, 1:1000) overnight at 4 °C. Membranes were then washed with TBS-T and incubated with appropriate species-specific HRP-conjugated secondary antibodies (Dako, 1:1000). Signals were visualised using Enhanced Chemiluminescence (ECL) reagents (Amersham) and exposure to film. Films were developed in a Konica Film Processor (SRX-101A). All blots were processed in parallel and derive from the same experiment.

### Immunofluorescence

Cells were fixed in 4% formaldehyde for 10 min and permeabilised with 0.1% Triton X-100 in PBS for 5 min. Cells were then blocked with 5% BSA in PBS for 10 min prior to incubation with primary antibodies specific for integrin β6 (Merck, 10D5, 1:100) and fibronectin (FN, Sigma, IST-4, 1:100) overnight at 4 °C. Cells were then washed with PBS and incubated with goat anti-mouse Alexa-Fluor 488 secondary antibody (Invitrogen, 1:200), followed by additional washing and then mounted and counterstained with ProLong Gold Antifade reagent containing 4′,6-diamidino-2-phenylindole (DAPI, Invitrogen). Images were viewed on a Zeiss LSM 710 Meta microscope.

### Quantitative reverse transcriptase-PCR (qRT-PCR) analysis

Total RNA was isolated from cells using the Quick-RNA MiniPrep Kit (Zymo Research). cDNA was synthesized using Moloney-Murine Leukemia Virus (M-MLV) reverse transcriptase with random nucleotide primers (Sigma). Quantitative reverse transcriptase-PCR (qRT-PCR) was performed on cDNA using SYBR Green Master Mix (Thermo Fisher Scientific) on a StepOnePlus Real-Time PCR System (Applied Biosystems). Gene expression was quantified using the following primers: 18 S forward: CACGGGAAACCTCACCCGGC; 18 S reverse: AACGGCCATGCACCACCACC; ITGB6 forward: GAAGGAATGATCACGTACAAG; ITGB6 reverse: AGCAGGGAGTCTTCACAGGT; FN1 forward: AACAAACACTAATGTTAATTGCCC; FN1 reverse: TCGGGAATCTTCTCTGTCAGC; TGFB1 forward: GGAAATTGAGGGCTTTCGCC; TGFB1 reverse: CCGGTAGTGAACCCGTTGAT; MMP9 forward: GAACCAATCTCACCGACAGG; MMP9 reverse: GCCACCCGAGTGTAACCATA; MMP13 forward: TCTACACCTACACCGGCAAA; MMP13 reverse: GGTTGGGGTCTTCATCTCCT. Fold changes in mRNA expression were calculated by the ΔΔCt method using 18 S as an endogenous control. Results are expressed as fold change by normalizing to the controls.

### Human Proteome Protease Array

Human proteome protease arrays (R&D Systems) were processed according to the manufacturer’s instructions using concentrated CM (250 μg). Signals were visualised using ECL reagents and exposure to film. Films were developed in a Konica Film Processor.

### Zymography

Concentrated CM (100 μg) was resolved on a 10% Tris-Glycine gel supplemented with 0.1% gelatin (Invitrogen). Gels were renatured in a buffer (2.5% (v/v) Triton X-100), followed by incubation in a developing buffer (5 mM Tris-Base, 4 mM HCl, 20 mM NaCl, 0.5 mM CaCl_2_) overnight at 37 °C and visualised with Coomassie R-250 (Thermo Fisher Scientific). Images of stained gels were captured under illumination using the UVP Imagestore 5000 (Ultra-Violet Products).

### Mechanical Stretch

Cells were seeded in flexible-bottomed BioFlex culture plates coated with type IV collagen (Dunn Lab), and grown for 72 h. Immediately prior to stretching, cells were removed from the periphery of the well, and the media was replaced. Cells were then exposed to a static stretch with 10% elongation of the flexible surface using a computerised vacuum-operated instrument (Flexcell strain unit FX-5000 Tension Plus; Flexcell International) maintained in a cell culture incubator, for 48 h. Unstretched controls cells were plated on BioFlex culture plates for an equivalent time but were not subjected to stretch.

### Statistical Analysis

GraphPad Prism (version 9.1.2) was used to perform all statistical analyses and statistical significance was determined using the appropriate tests as noted in the corresponding figure legends.

## Supplementary information

Supplementary Information file

## Data Availability

The array dataset analysed to assess expression profiles changes in human breast tissues is available under accession number GSE21422^42^. All further datasets generated and analysed in this study are available from the corresponding author upon reasonable request.
