# Supplementary material for: Mechanostimulation of breast myoepithelial cells induces functional changes associated with DCIS progression to invasion
Source: NPJ Breast Cancer. 2022 Sep 20;8:109. doi: 10.1038/s41523-022-00464-4 (PMC9489768; doi:10.1038/s41523-022-00464-4)
Supplement: Supplementary file 1 — Supplementary Information file [file 41523_2022_464_MOESM1_ESM.pdf]

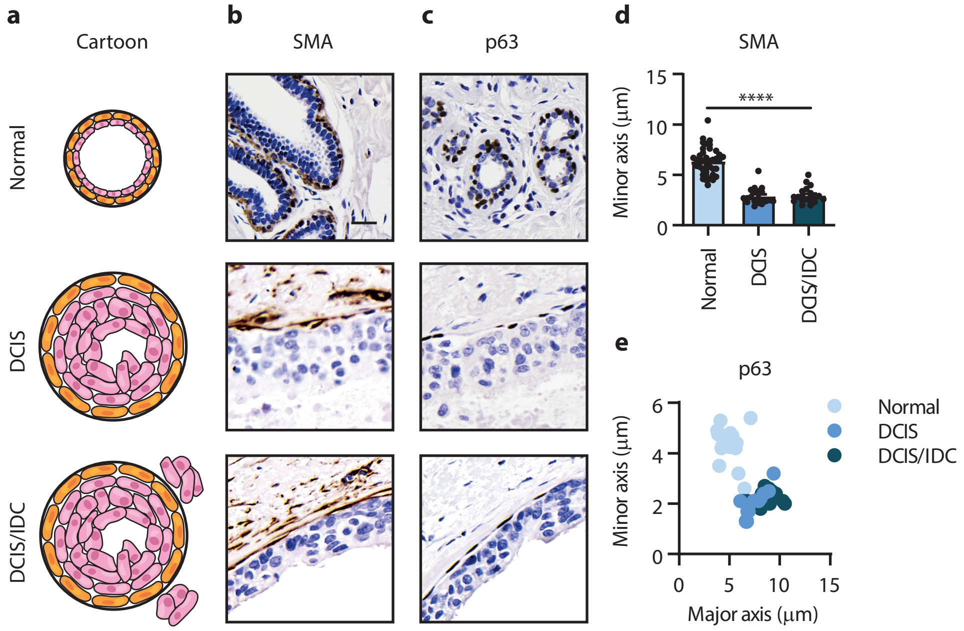

Supplementary Figure 1. Morphological changes to myoepithelial cells in DCIS. a, Cartoon depicting a normal duct, a DCIS duct without (DCIS) and with co-existent invasion (DCIS/IDC). **b**,**c**, Representative images of smooth-muscle actin (SMA) (**b**) and p63 (**c**) by immunohistochemical staining in human breast tissue samples featuring adjacent normal ducts, DCIS and DCIS/IDC. **d**, Bars showing the mean minor axis of myoepithelial cells in adjacent normal (n = 40), DCIS (n = 20) and DCIS/IDC (n = 20) patient samples (error bars, +s.e.m). ****$P < 0.0001$ (Kruskal–Wallis one-way ANOVA). **e**, Dots represent the mean major and minor axis of myoepithelial cell nuclei in adjacent normal, DCIS and DCIS/IDC patient samples. Each dot represents an individual patient sample (n = 40).

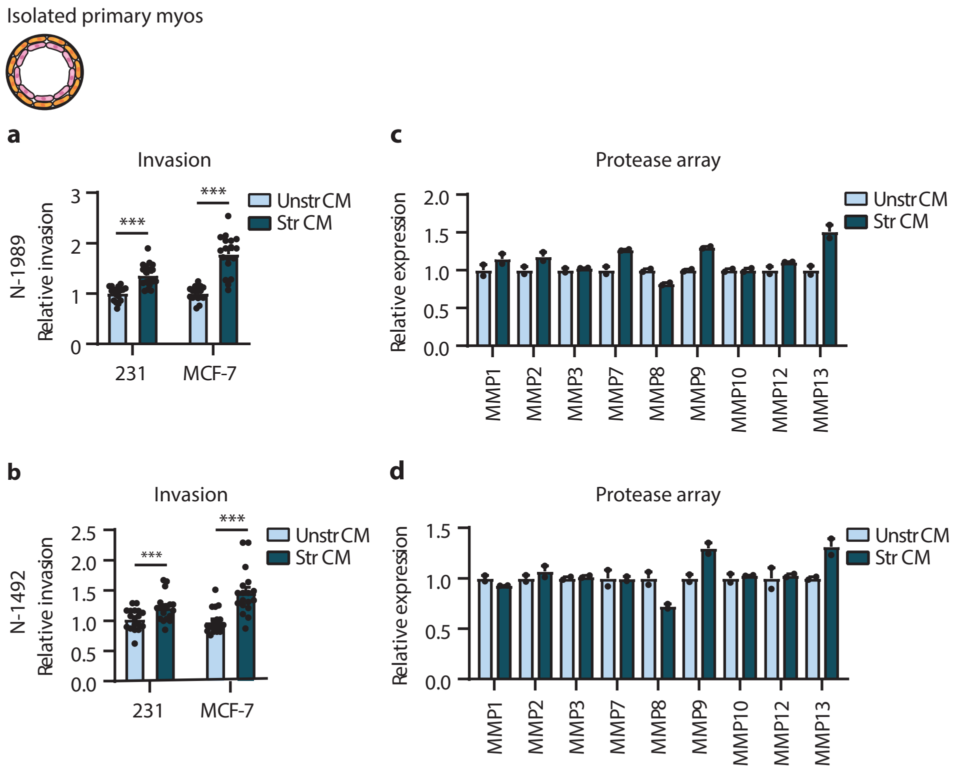

Supplementary Figure 2. Mechanical stretching primary normal myoepithelial cells activates a tumour promoting phenotype. **a**,**b**, Invasion assay for MDA-MB-231 (231) and MCF-7 cells using conditioned media (CM) isolated from unstretched (Unstr) or stretched (Str) primary normal myoepithelial cells; N-1989 (**a**) and N-1492 (**b**) (error bars, +s.e.m). n = 3 biological replicates, 4-6 technical replicates; MDA-MB-231 ***P = 0.0001 (two-tailed t-test). **c**,**d**, Array analysis for proteases using conditioned media isolated from unstretched or stretched myoepithelial cell line; N-1989 (**c**) and N-1492 (**d**) (error bars, +s.e.m). n = 1 biological replicate, 2 technical replicates.

Full unedited blots for Figure 2d

Left panel

Right panel

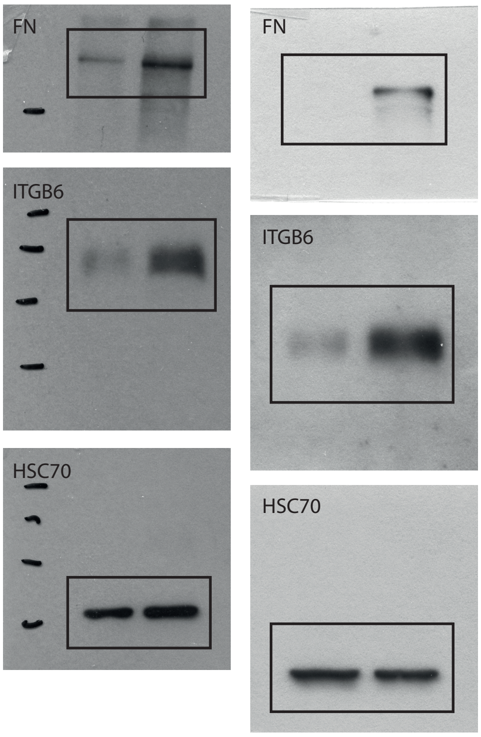

Full unedited blots for Figure 2f

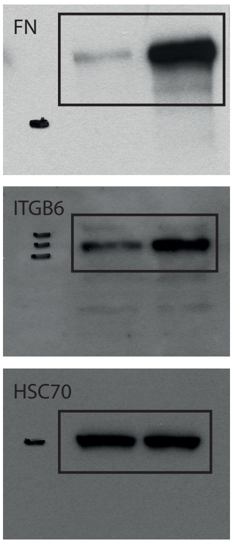

Full unedited blots for Figure 3d

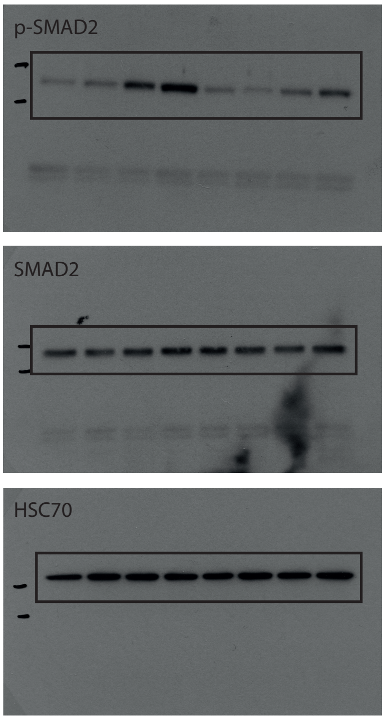

Full unedited blots for Figure 2g

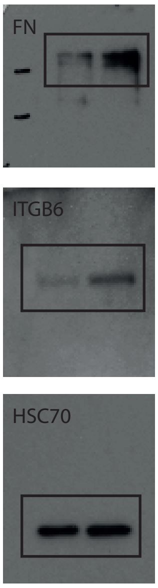

Full unedited blots for Figure 2h

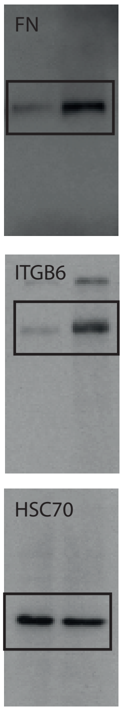

Full unedited blots for Figure 2i

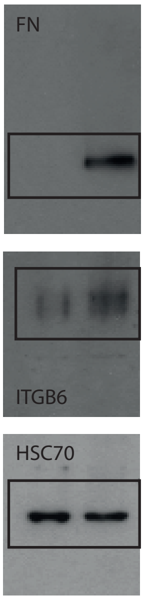

Full unedited blot for Figure 6c-e

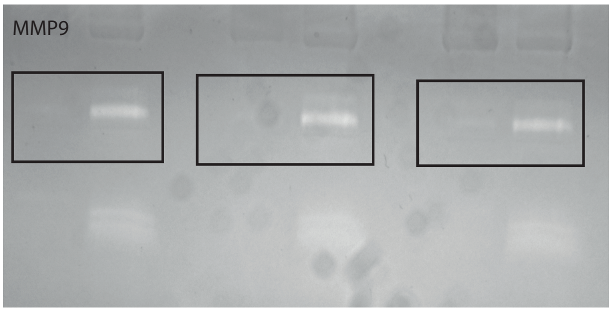

Full unedited blots for Figure 7b

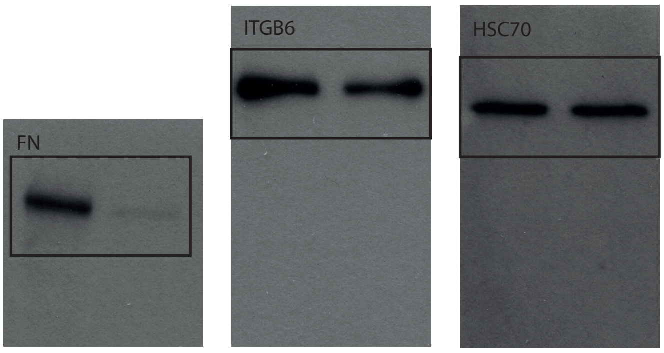

Supplementary Figure 3. Unprocessed immunoblot images related to the indicated blots.

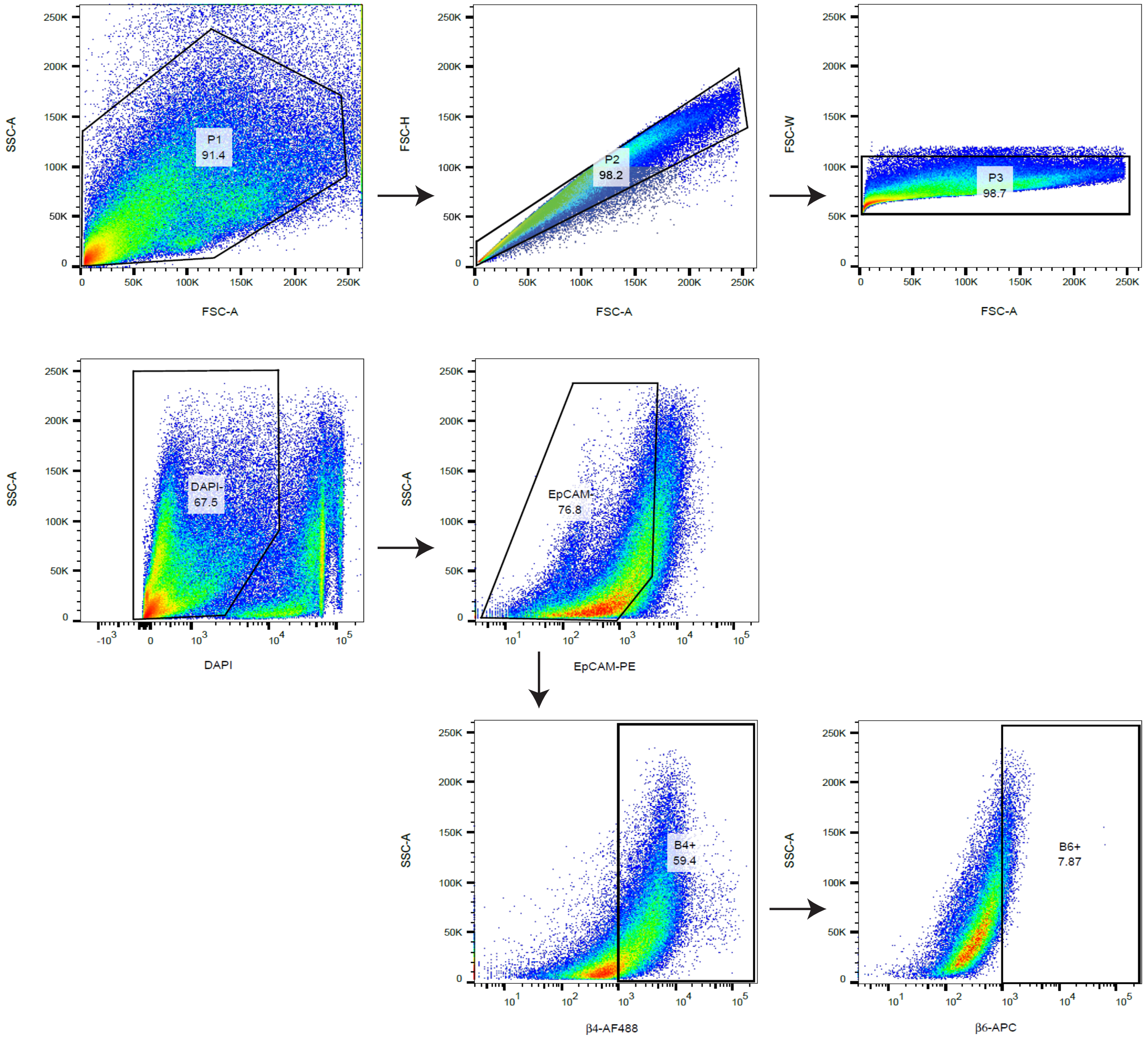

Supplementary Figure 4. Gating strategy for FACS of DCIS-associated myoepithelial cells.

Supplementary Table 1: Clinical annotation of DCIS samples analysed by IHC

Features	DCIS	DCIS/IDC
Age (mean ± SD)	54.75 ± 4.51	55.55 ± 3.15
>55	10	12
<55	10	8
Grade		
Low	10	
High	10	20
ER status		
Positive	12	14
Negative	8	5
Not processed		1
PR status		
Positive	8	10
Negative	12	7
Not processed		3
HER2 status		
Positive	1	2
Negative		16
Not processed	19	2

Supplementary Table 2: Myoepithelial cell expression of integrin β6 in DCIS samples

	Number of ducts (%)		
	Positive	Negative	Total
Normal	0 (0%)	944 (100%)	944
DCIS			
Non-high grade	165 (27%)	456 (74%)	621
High grade	256 (45%)	313 (55%)	569
DCIS/IDC	473 (68%)	224 (32%)	697
			2831

Supplementary Table 3: Periductal fibronectin expression in DCIS samples

	Number of ducts (%)		
	Positive	Negative	Total
Normal	57 (6%)	864 (94%)	921
DCIS			
Non-high grade	432 (70%)	188 (30%)	620
High grade	364 (66%)	186 (34%)	550
DCIS/IDC	556 (87%)	82 (13%)	638
			2729

Supplementary Table 4: Expression of myoepithelial cell integrin β6 and periductal fibronectin in DCIS samples

	Number of ducts (%)				
	β6-pos/FN-pos	β6-pos/FN-neg	β6-neg/FN-pos	β6-neg/FN-neg	Total
DCIS					
Non-high grade	139 (23%)	17 (3%)	267 (45%)	170 (29%)	593
High grade	226 (42%)	21 (4%)	166 (30%)	131 (24%)	544
DCIS/IDC	345 (63%)	9 (2%)	134 (24%)	60 (11%)	548
					1685

Supplementary Table 5: MMP13 expression in DCIS samples

	Number of ducts (%)		
	Positive	Negative	Total
Normal	0 (0%)	775 (100%)	775
DCIS			
Non-high grade	179 (33%)	365 (67%)	544
High grade	274 (48%)	301 (52%)	575
DCIS/IDC	416 (72%)	161 (28%)	577
			2471

Supplementary Table 6: DCIS duct size in relation to myoepithelial cell expression of integrin β6

	Duct diameter in μm2 (number of ducts)		
	Positive	Negative	Total
Normal		40 (420)	420
DCIS			
Non-high grade	340 (125)	309 (233)	358
High grade	470 (235)	430 (238)	473
DCIS/IDC	476 (353)	415 (185)	538
			1789
